# A rationale for considering heart/brain axis control in neuropsychiatric disease

**DOI:** 10.1007/s00335-022-09974-9

**Published:** 2022-12-20

**Authors:** Lillian Garrett, Dietrich Trümbach, Nadine Spielmann, Wolfgang Wurst, Helmut Fuchs, Valerie Gailus-Durner, Martin Hrabě de Angelis, Sabine M. Hölter

**Affiliations:** 1 German Research Center for Environmental Health, Institute of Experimental Genetics and German Mouse Clinic, Helmholtz Zentrum München, Neuherberg, Germany; 2 German Research Center for Environmental Health, Institute of Developmental Genetics, Helmholtz Zentrum München, Neuherberg, Germany; 3 Chair of Developmental Genetics, TUM School of Life Sciences, Technische Universität München, Freising-Weihenstephan, Germany; 4 Deutsches Institut Für Neurodegenerative Erkrankungen (DZNE) Site Munich, Feodor-Lynen-Str. 17, 81377 Munich, Germany; 5 Munich Cluster for Systems Neurology (SyNergy), Adolf-Butenandt-Institut, Ludwig-Maximilians-Universität München, Feodor-Lynen-Str. 17, 81377 Munich, Germany; 6 Chair of Experimental Genetics, TUM School of Life Sciences, Technische Universität München, Alte Akademie 8, 85354 Freising, Germany; 7 German Center for Diabetes Research (DZD), Ingolstädter Landstr. 1, 85764 Neuherberg, Germany; 8 Technische Universität München, Freising-Weihenstephan, Germany; 9 Helmholtz Center Munich, Institute of Developmental Genetics, Ingolstädter Landstr. 1, 85764 Neuherberg, Germany; 10 German Research Center for Environmental Health, Institute of Metabolism and Cell Death, Helmholtz Zentrum München, Neuherberg, Germany

## Abstract

Neuropsychiatric diseases (NPD) represent a significant global disease burden necessitating innovative approaches to pathogenic understanding, biomarker identification and therapeutic strategy. Emerging evidence implicates heart/brain axis malfunction in NPD etiology, particularly via the autonomic nervous system (ANS) and brain central autonomic network (CAN) interaction. This heart/brain inter-relationship harbors potentially novel NPD diagnosis and treatment avenues. Nevertheless, the lack of multidisciplinary clinical approaches as well as a limited appreciation of molecular underpinnings has stymied progress. Large-scale preclinical multi-systemic functional data can therefore provide supplementary insight into CAN and ANS interaction. We here present an overview of the heart/brain axis in NPD and establish a unique rationale for utilizing a preclinical cardiovascular disease risk gene set to glean insights into heart/brain axis control in NPD. With a top-down approach focusing on genes influencing electrocardiogram ANS function, we combined hierarchical clustering of corresponding regional CAN expression data and functional enrichment analysis to reveal known and novel molecular insights into CAN and NPD. Through ‘support vector machine’ inquiries for classification and literature validation, we further pinpointed the top 32 genes highly expressed in CAN brain structures altering both heart rate/heart rate variability (HRV) and behavior. Our observations underscore the potential of HRV/hyperactivity behavior as endophenotypes for multimodal disease biomarker identification to index aberrant executive brain functioning with relevance for NPD. This work heralds the potential of large-scale preclinical functional genetic data for understanding CAN/ANS control and introduces a stepwise design leveraging preclinical data to unearth novel heart/brain axis control genes in NPD.

**Supplementary Information:**

The online version contains supplementary material available at 10.1007/s00335-022-09974-9.

## Introduction

Neuropsychiatric diseases (NPD) are clinically heterogeneous and comprise 7% of the global disease burden (Rehm and Shield 2019; Yang et al. 2021). Cardiovascular disease (CVD) is similarly prevalent (Roth et al. 2020) and a significant cause of premature death in NPD (Correll et al. 2017) inferring pathogenic overlap. Poor lifestyle leading to obesity and diabetes as well as medication use partially underlie CVD/NPD comorbidity (Correll et al. 2015; Hamer et al. 2008; Penninx and Lange 2018; Rosengren et al. 2004). Nevertheless, heart and brain miscommunication through the heart/brain axis likely contributes to pathogenesis. Particularly the NPD implications of autonomic nervous system (ANS) dysfunction attracted some attention (Mulkey and du Plessis 2019; Perrone and Valente 2021). The brain’s central autonomic network (CAN), via the ANS, transduces emotional and psychological experience effects on heart activity with abnormalities therein increasing NPD and comorbid CVD risk (Mulkey and du Plessis 2019). The prevalence and therapeutic shortcomings highlight the need for more innovative approaches to tackling NPDs. Thus, a deeper understanding of this bidirectional brain/heart relationship, including the trans-diagnostic potential of heart rate variability (HRV), will evince potentially unexplored NPD biomarkers, endophenotypes (genetic dysfunction traits), and risk gene variants for precision psychiatry approaches (Beauchaine and Thayer 2015).

An impediment to heart/brain axis understanding in NPD and neurocardiology is the dearth of holistic approaches to patient treatment and the current emphasis on specialization in one medical field (Valenza et al. 2016). The availability of large-scale preclinical multi-systemic data is thus a valuable resource to gain insights into the molecular mechanism of heart/brain interaction for patient translation. In this report, we provide, therefore, an introduction to heart/brain axis and CAN/ANS communication in NPD. Moreover, we outline a unique rationale and stepwise refinement incorporating brain expression data of a previously identified CVD risk gene set from the International Mouse Phenotyping Consortium (IMPC) data (Spielmann et al. 2022). By addressing NPD understanding from a CVD risk perspective, we introduce a novel means of exploiting preclinical multi-disciplinary data to prioritize NPD candidate genes and unearth unrealized molecular foundations of heart/brain and CAN/ANS abnormality as a blueprint for innovative NPD therapies.

## Heart/brain abnormality in NPD—the evidence from CVD and NPD comorbidity data

Epidemiological studies indicate an upsurge in NPD prevalence in the last 20 years, particularly in early-onset diseases such as autism spectrum disorder (ASD) and attention deficit hyperactivity disorder (ADHD) (Atladottir et al. 2015, 2007; Kim et al. 2011; Maenner et al. 2021). The increase likely relates to non-etiological features (e.g. broadened diagnostic criteria) and additional factors (parental lifestyle, advancing age at conception) increasing abnormal genetic neurodevelopmental risk. NPDs exhibit moderate to high heritability particularly ASD, schizophrenia (SCZ) and bipolar disorder (BD), and major depressive disorder (MDD) to a lesser extent (Faa et al. 2016; Sandin et al. 2017). In ASD, much of the genetic risk originates from common variants, as well as rare and de novo variants with gene x environment (GxE) interactions and epigenetics accounting for missing heritability. Such elements are critical during intrauterine and postnatal neurodevelopment when there is greater vulnerability to adverse early life events and developmental influences (Faa et al. 2016).

While neurocardiology is still a burgeoning field, there is evidence connecting NPD and CVD pathogenesis that reflects the so far established bidirectional heart/brain interaction. Depression symptoms, for example, are frequent in coronary artery disease patients [particularly in young and female patients (Shah et al. 2014)]. In addition, heart failure can impair cerebral perfusion, leading to stroke, cognitive obstruction and mood disorder [reviewed by (Doehner et al. 2018)]. Among NPDs, MDD, anxiety disorders, SCZ and ASD are comorbid with cardiovascular dysfunction (Kawachi et al. 1994; Lett et al. 2004; Tyler et al. 2011; Westman et al. 2018). For example, both MDD and SCZ patients are at increased coronary heart disease risk while SCZ patients are also vulnerable to cerebrovascular disease and congestive heart failure (Correll et al. 2017; Rugulies 2002). Furthermore, severe anxiety and stress experience are associated with sudden cardiac death and Takotsubo (“broken heart”) cardiomyopathy related to sympathomimetic overflow (Doehner et al. 2018; Watkins et al. 2006). Congenital heart disease (CHD) is also a risk factor for neurodevelopmental disorders such as ASD (Wernovsky and Licht 2016) potentially related to shared genetic origins and/or vulnerability to environmental challenges during development.

Additional, likely overlapping, bidirectional brain/heart communication channels underlying CVD and NPD risk include the hypothalamic–pituitary–adrenal (HPA) axis, inflammatory signaling and ANS dysfunction (De Hert et al. 2018). HPA-induced cortisol hyperactivity in MDD augments coronary artery atherosclerosis and cardiovascular death risk (Jokinen and Nordstrom 2009; Lichtman et al. 2008; Vale 2005). Furthermore, inflammation and increased cytokine levels [interleukin (IL)-6, IL-1β, tumor necrosis factor (TNF)-α] are etiological in both NPD and CVD (Empana et al. 2005). Disrupted CAN and ANS development impair stress resilience increasing NPD vulnerability (Homsy et al. 2015). Thus, understanding the genetic underpinnings of heart/brain axis interaction, particularly via the ANS and CAN, has the potential to provide so far unexploited disease and patient stratification biomarkers as well as NPD therapeutic avenues. As the Research Domain Criteria (RDoC) initiative seeks objective NPD markers (Insel et al. 2010), such novel endophenotype identification is welcome.

## An overview of heart/brain communication via CAN—feedforward and feedback ANS control

**Fig. 1:**
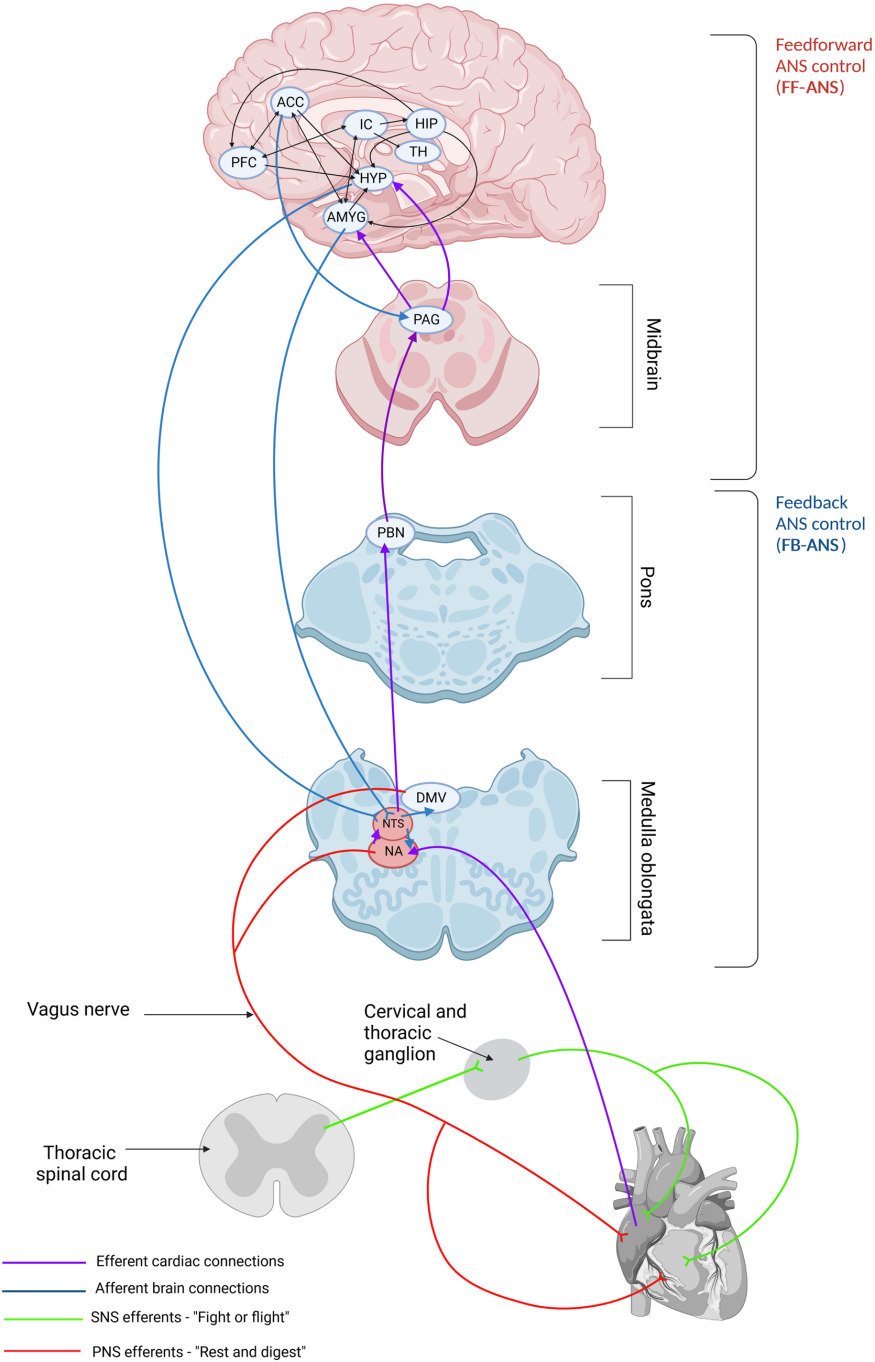
A highly schematized representation of the complex central autonomic network (CAN) organization in the brain involved in both feedforward [FF-ANS (red shading)] and feedback [FB-ANS (blue shading)] control of autonomic nervous system (ANS) function and the neuroanatomical correlate of heart/brain axis communication. The most important CAN brain structures include the prefrontal cortex (PFC), anterior cingulate cortex (ACC), insular cortex (IC), hippocampus (HIP), amygdala (AMYG), thalamus (TH), hypothalamus (HYP) as well as periaqueductal gray (PAG) in the midbrain, parabrachial nucleus (PBN) of the pons and the following medulla oblongata nuclei: dorsal motor vagus (DMV), solitary tract (NTS) and ambiguous (NA). This network of brain regions interconnects as depicted and, via sympathetic (SNS) and parasympathetic (PNS) connections, influence heart function. Parasympathetic output extends from the DMV to NA projecting through the vagus nerve to the heart sino-atrial node (Created with Biorender.com)

The complex nature of CAN and ANS interaction and function warrants an overview to understand NPD-related dysfunction. The ANS modulates the intrinsic cardiac nervous system, sinoatrial and atrioventricular node activity, transducing peripheral signals (autonomic afference) into physiological responses (autonomic efference). The efferent system dichotomizes into sympathetic (SNS) and parasympathetic (PNS) branches (Fig. 1) (Karemaker 2017). SNS preganglionic neurons from the thoracic spinal cord synapse with neurons in cervical and thoracic ganglia and postganglionic fibers innervate the cardiac conduction system (Silvani et al. 2016). Preganglionic PNS neurons originate from the nucleus ambiguous and dorsal motor nucleus of the vagus in the medulla oblongata. Via the vagus nerve, these neurons extend to the cardiac nervous system and postganglionic PNS fibers innervate the cardiac conduction system (Silvani et al. 2016). “Fight or flight” emergency responses to stress and danger, including increased heart rate (HR), are mediated by the SNS and the PNS underlies “rest and digest” restorative functions. Preganglionic SNS and postganglionic PNS nerve endings release acetylcholine while postganglionic SNS terminals release predominantly noradrenaline (Waxenbaum et al. 2022). Autonomic imbalance occurs with hyper-activation of one ANS branch, usually the SNS, at the expense of the other (PNS) augmenting pathological risk and even death (arrhythmias, sudden cardiac death). Low HRV signifies decreased vagal tone and is a non-invasive disease marker in this case (Cerritelli et al. 2021).

The CAN encompasses brain cortical and subcortical mood and memory influences on ANS cardiovascular activity, dividing into ‘feedforward’ (FF-ANS) and ‘feedback’ (FB-ANS) brain regions (Fig. 1) (Dampney 2016). The former includes the insular and anterior cingulate (ACC) cortices, amygdala, hypothalamus and the periaqueductal gray (PAG) region (Scheitz et al. 2020; Silvani et al. 2016). These brain regions alter ANS (both SNS and PNS) and HPA axis activity to regulate cardiovascular function (e.g. HR) (Dampney 2016). The insular cortex is a viscerosensory and -motor brain area (Nagai et al. 2010), highly connected to the cortex, thalamus, amygdala and hippocampus. External somatosensory information transmits to the agranular insula integrating with cardiac afferent input received by the posterior granular and mid-dysgranular insular cortex (Ceunen et al. 2016; Nieuwenhuys 2012). Along with ACC and amygdala, the insula mediates interoception i.e. the awareness of internal bodily states such as cardiac palpitations in panic disorder (Craske et al. 2010; Khoury et al. 2018). The ACC (Gillies et al. 2019) connects to the hypothalamus and PAG and associates with sympathetic modulation of HR (Critchley et al. 2003). The PAG itself encapsulates the midbrain cerebral aqueduct connecting descending and ascending projections concerning emotion-related and motor information (Vianna and Brandao 2003). The hippocampus and amygdala connect to the hypothalamic paraventricular nucleus (PVN) of the HPA axis and respond to cortisol through glucocorticoid receptors. Both regions are involved in memory and mood, the former particularly in fear memory. The thalamus and cortex connect to the central amygdala in response to stressful experiences and the parvocellular PVN neurons adjust the SNS outflow to the heart facilitating a “fight or flight” response, (Ferguson et al. 2008).

Feedback control involves brainstem ANS nuclei mediating reflex effects in response to peripheral receptor input (Fig. 1). The medullary nucleus of the solitary tract (NTS) receives baroreflex information concerning cardiovascular feedback regulation controlling arterial pressure. NTS activation decreases blood pressure and HR. It is highly connected to the pons parabrachial nucleus that transmits cardiovascular information to forebrain regions increasing SNS activity and tachycardia (Davern 2014). Feedback control includes also the aforementioned nucleus ambiguous from where PNS vagal cardiac outflow stems (Dampney 2016).

## Evidence implicating abnormal CAN and ANS in NPD

The neuroanatomical organization of the CAN permits psychological states to influence cardiovascular ANS control via or even bypassing the brainstem homeostatic nuclei. Thus, HRV, the HR beat-to-beat variability measured in an electrocardiogram (ECG), is a valuable non-invasive ANS index in humans and animal models (Laborde et al. 2017; Thayer and Lane 2000; Thireau et al. 2008). Given this and the high heritability of this trait, it has potential as an NPD endophenotype (Golosheykin et al. 2017). Thus, we here describe several, albeit at times controversial, lines of evidence connecting HRV and hence abnormal ANS in anxiety/post-traumatic stress disorder (PTSD), MDD and SCZ.

In terms of anxiety, laboratory stressors increase SNS and decrease PNS cardiovascular control with fight-or-flight activation (Berntson et al. 1994; Pagani et al. 1991; Sloan et al. 1996). This state manifests as increased low frequency (LF)-HRV (reflecting baroreceptor reflex modulation and both SNS and PNS activity) and decreased high frequency (HF)-HRV (reflecting PNS activity) power or an increased LF/HF ratio (Goldstein et al. 2011; Reyes del Paso et al. 2013). Consistent with autonomic inflexibility, evidence indicates decreased HF-HRV across several anxiety disorders including panic disorder (PD), generalized anxiety disorder (GAD), social anxiety and obsessive–compulsive disorder (OCD) (Chalmers et al. 2014; Pittig et al. 2013). The findings for PTSD have been less consistent however, limited by small sample sizes with diverse individual patient experience (Dennis et al. 2014; Ge et al. 2020). Abnormal function of CAN brain regions mediating executive function likely underlies the associated aberrant HRV (Lee et al. 2022). While the amygdala activates in fear conditioning (LaBar et al. 1998) transmitting brief exposure information, the more general anxiety states require the medial PFC input to maintain protracted attentional focus on threat-related cues (Eippert et al. 2007).

MDD is generally associated with decreased HF-HRV (Jangpangi et al. 2016; Koch et al. 2019; Moretta and Messerotti Benvenuti 2022) yet the relationship between ANS function and disease severity is complex (Sarlon et al. 2021). A deeper understanding of this connection is particularly germane given that MDD is a risk for CVD (Correll et al. 2017). The pathological origin of altered HRV in MDD is not clear although comorbid anxiety disorder may be partially responsible. Depressive states are associated with obstructed communication between ACC and brainstem ANS nuclei (Critchley et al. 2005; Makovac et al. 2015; Ruiz-Padial et al. 2011). In addition, depression severity correlates with vagal tone loss highlighting the value of HRV as an MDD metric for which vagal nerve stimulation is a proposed treatment (O'Reardon et al. 2006). Takotsubo cardiomyopathy further implicates severe anxiety and depressive states and is associated with reduced thickness of the insula and ACC (Hiestand et al. 2018).

The data available for HRV in SCZ is less consistent although of immense potential. Thayer and Lane (Thayer and Lane 2000) proposed a so-called Neurovisceral Integration Model to denote the utility of HRV as a proxy index of PFC function and cognitive ability. The PFC inhibits SNS activation thereby augmenting vagal tone and altered PFC function directly reflects in HRV measures. Specifically, HF-HRV aligns with increased blood flow to the PFC and ACC (Thayer et al. 2012). These brain regions mediate working memory, executive control of attentional shifting, decision-making and sociability that are impaired in SCZ (Morris et al. 2013). In general, high HRV is associated with improved executive functioning (Hansen et al. 2004) as well as superior stress resilience with low HRV, the opposite (Pulopulos et al. 2018). HRV decreased in SCZ patients correlating with symptom severity thereby emphasizing the benefit as a dependent variable in cognitive analysis (Morris et al. 2013). In general, despite this available information, supporting evidence is limited and gaps remain in understanding the genetic underpinnings of CAN-based ANS and HRV control and disease risk.

## Leveraging large-scale preclinical functional genetic data to gain insight into CAN/ANS in NPD

A promising approach to identify new disease biomarkers and therapeutic targets is to pin-point novel candidate genes that, when mutated, cause abnormal CAN and ANS activity augmenting NPD risk. While the lack of holistic clinical interdisciplinary data impedes heart/brain axis understanding (Valenza et al. 2016), the Diagnostic and Statistical Manual of Mental Disorders (DSM-5) nonetheless advocates for integrated psychiatric patient care considering medical and mental conditions in parallel (American Psychiatric Association 2022). As a move in this direction, the IMPC resource (Brown and Moore 2012) provides open access to large-scale multi-systemic experimental data from single knockout mouse lines generated for each protein-coding gene in the genome [see www.mousephenotype.org, 8916 genes annotated to date, Data Release (DR) Version 17.0, (Brown and Moore 2012)]. Utilizing data contained therein can reveal molecular mechanisms not easily detectable in a clinical setting, especially in the case of brain disease. It is thus a valuable tool to understand heart/brain interaction in NPD.

We introduce here a rationale for distillation of this large-scale data to prioritize candidate genes involved in heart/brain axis control. Recently, Spielmann et al. (2022) exploited IMPC cardiovascular data (3894 single-gene null deletion alleles) to establish 705 CVD risk genes, associated with ECG and/or trans-thoracic echocardiography (TTE) phenotypes in young adult mice (12-weeks of age). This included 486 genes with no former cardiac dysfunction association causing cardiac rhythm disorder, cardiomyopathies, or structural heart defects. Given that ECG parameters (HR/HRV) potentially index CAN and PFC function, and that a similar parameter and diagnostic can be applied to humans and mice, we implemented a unique stepwise refinement of this ECG gene set. The goal was to derive novel NPD insight from an initial CVD risk starting point. To this end, we capitalized on the assertion that analysis of gene expression can infer function for a range of tissues (Hume et al. 2010; Mabbott et al. 2013). Thus, combining hierarchical clustering of the corresponding ECG gene CAN regional brain expression data with functional enrichment analysis, we gathered and characterized candidate genes likely involved in modulating cardiovascular activity via the CAN. Furthermore, with subsequent ‘support vector machine’ inquiries and literature validation, we deduced 32 CAN-expressed genes altering HR/HRV for future NPD empirical validation.

### Stepwise design and methods applied

**Fig. 2:**
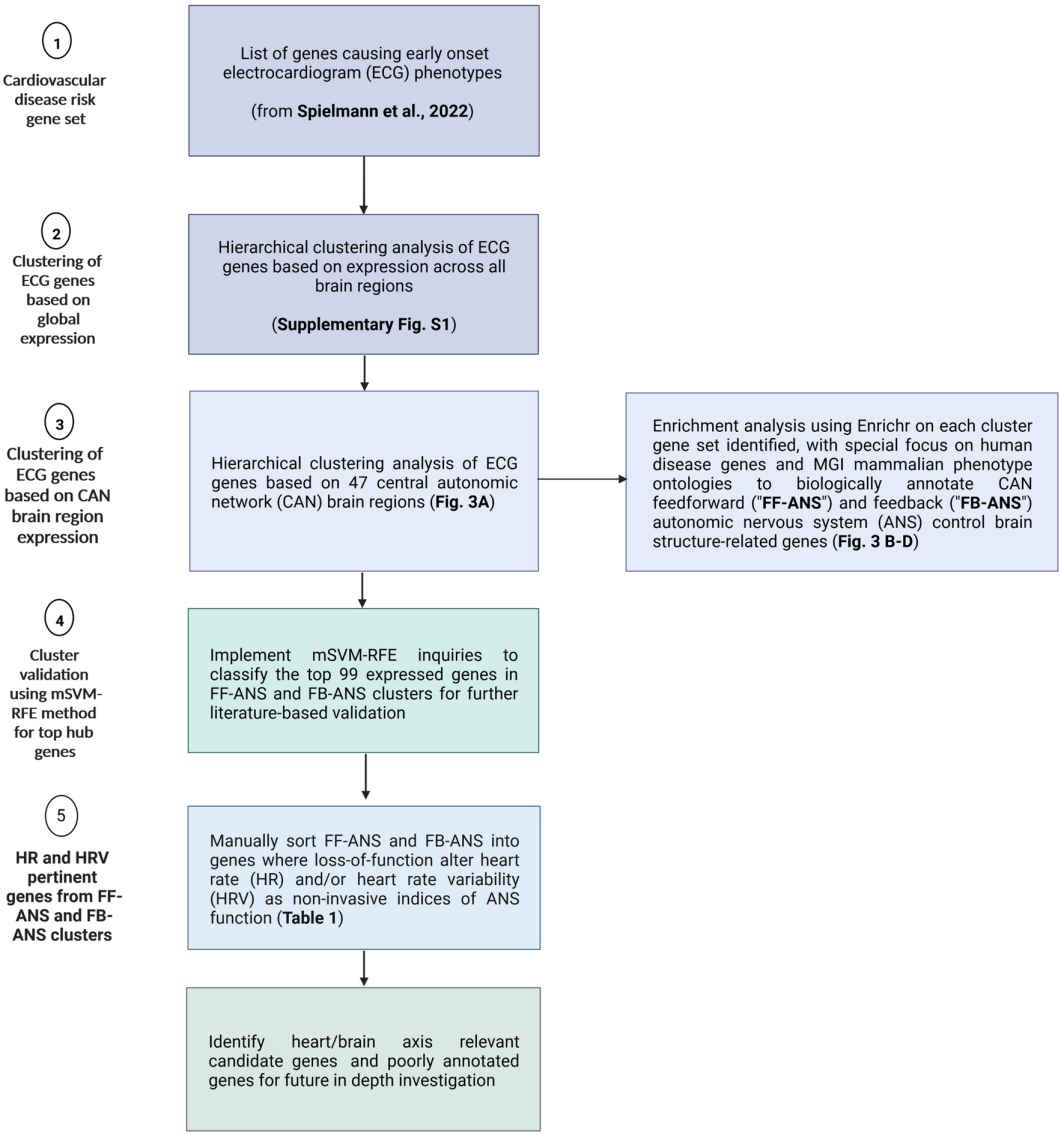
Shows the workflow and stepwise design used for characterization of the ECG gene set identified by (Spielmann et al. 2022). We first performed an unsupervised hierarchical clustering analysis of brain regions and gene expression data from 418 ECG genes, using all brain regions and then with 47 central autonomic network (CAN) relevant brain regions obtained from the Allen Brain Atlas. We then implemented Enrichr to perform gene set enrichment analysis on the two clusters of genes identified for functional annotation purposes. The two clusters were designated “FF-ANS” for feedforward and “FB-ANS” for feedback control to denote genes involved in either form of cardiovascular function in the CAN. We focused on human disease and Mammalian Phenotype annotations from the MGI database for validating our clustering algorithm. We used the mSVM-RFE method to identify the top 99 hub genes from all 418 genes. Using manual curation, we then focused on FF genes that caused heart rate (HR) or heart rate variability (HRV) phenotypes in mice to identify genes that influence ECG activity via the CAN and we earmarked poorly annotated genes as those potentially novel heart/brain axis genes in neuropsychiatric disease (Created with Biorender.com)

**Fig. 3:**
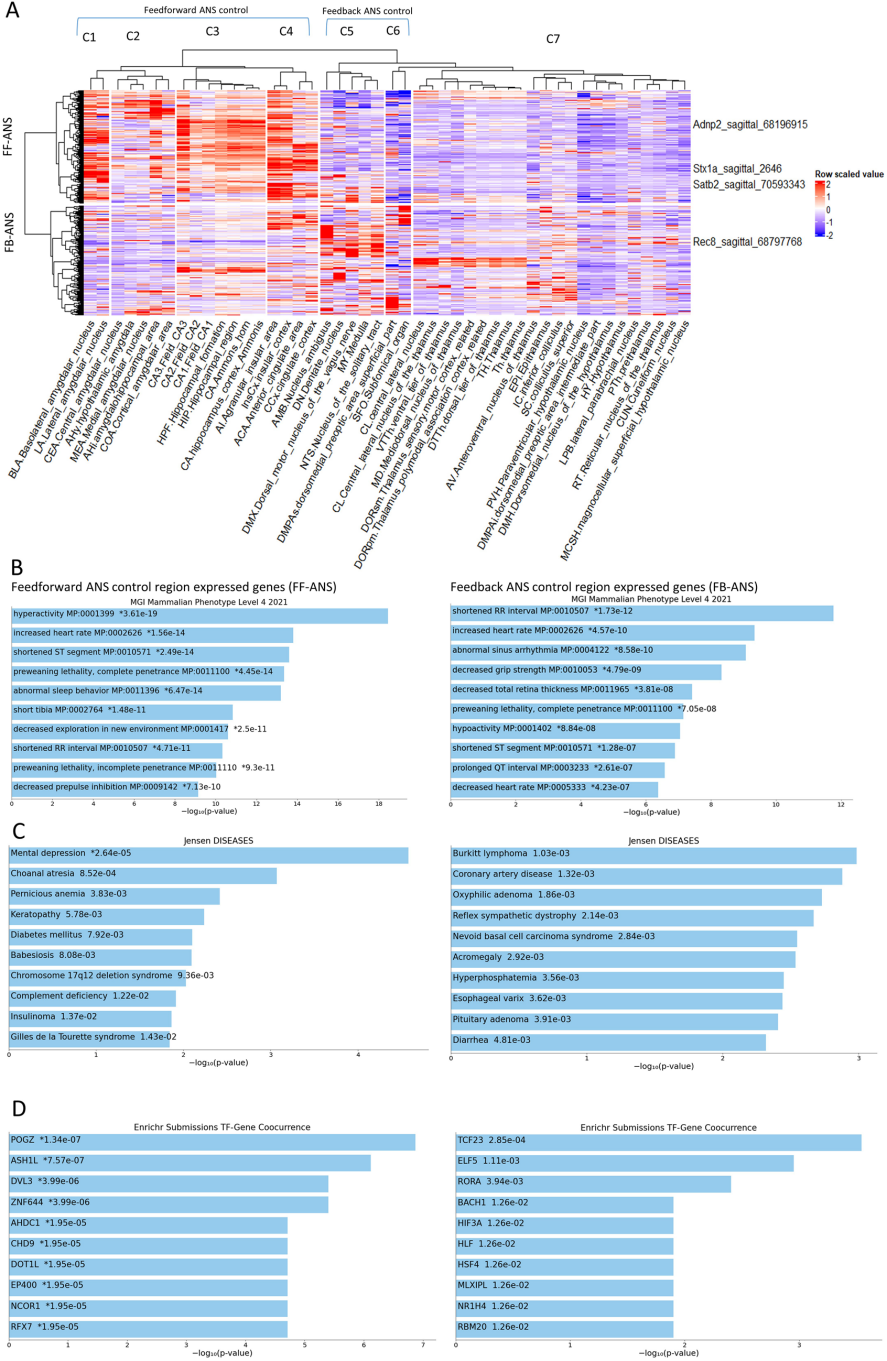
**A** Dendrogram and heat map of the unsupervised hierarchical clustering analysis of 47 central and brainstem autonomic nervous system (ANS) relevant brain regions and gene expression data from 418 electrocardiogram (ECG) genes. The columns each represent individual brain areas and the rows represent each gene (z-scores calculated across rows). There were 7 brain region clusters (C1-7) and 2 ECG gene clusters designated “FF-ANS” for feedforward and “FB-ANS” for feedback control of cardiovascular ANS function. Red and blue reflect up and downregulated expression levels, respectively, within each brain structure relative to all the other brain structures. **B-D** Bar charts of top enriched terms from the MGI Mammalian Phenotype terms level 4 2021, Jensen diseases and Enrichr submissions TF-gene co-occurrence gene set libraries respectively for feedforward (FF-ANS) and feedback (FB-ANS) control clusters. The top 10 enriched terms for the input gene set are displayed based on the -log10(*p*-value), with the actual *p*-value shown next to each term. The asterisk indicates significance related to the adjusted p-value. The term at the top has the most significant overlap with the input query gene set. The graphs were generated using the Enrichr interface: https://maayanlab.cloud/Enrichr/

A summary of our stepwise analysis design appears in Fig. 2. Spielmann et al. (2022) also detailed the 705 cardiovascular dysfunction genes identified (from IMPC DR10.1), the mouse lines used and the analyses performed. We focused on the ECG gene set and implemented mouse-brain in situ hybridization (ISH) raw values i.e. expression energies and corresponding brain tissues from the mouse Allen Brain Atlas (ABA) (Lein et al. 2007) that yielded this information for 2426 defined brain areas focusing on data from sagittal sections. Of the 462 genes associated with an ECG phenotype, we could obtain unique expression data for 418. Gene expression energy data of the whole mouse brain were downloaded via the Allen Brain Atlas application programming interface (API) (brain-map.org/api/index.html, following “Example Queries for Experiment Metadata”, database update March 04, 2021) by using custom-written bash scripts. We then performed a global unsupervised hierarchical clustering analysis, first focusing on all brain areas with all genes for each phenotype (Fig. S1) and then with CAN brain regions (Fig. 3A) as described previously [Feedforward (FF-ANS) and feedback (FB-ANS) control, (Dampney 2016)].

**Fig. 4:**
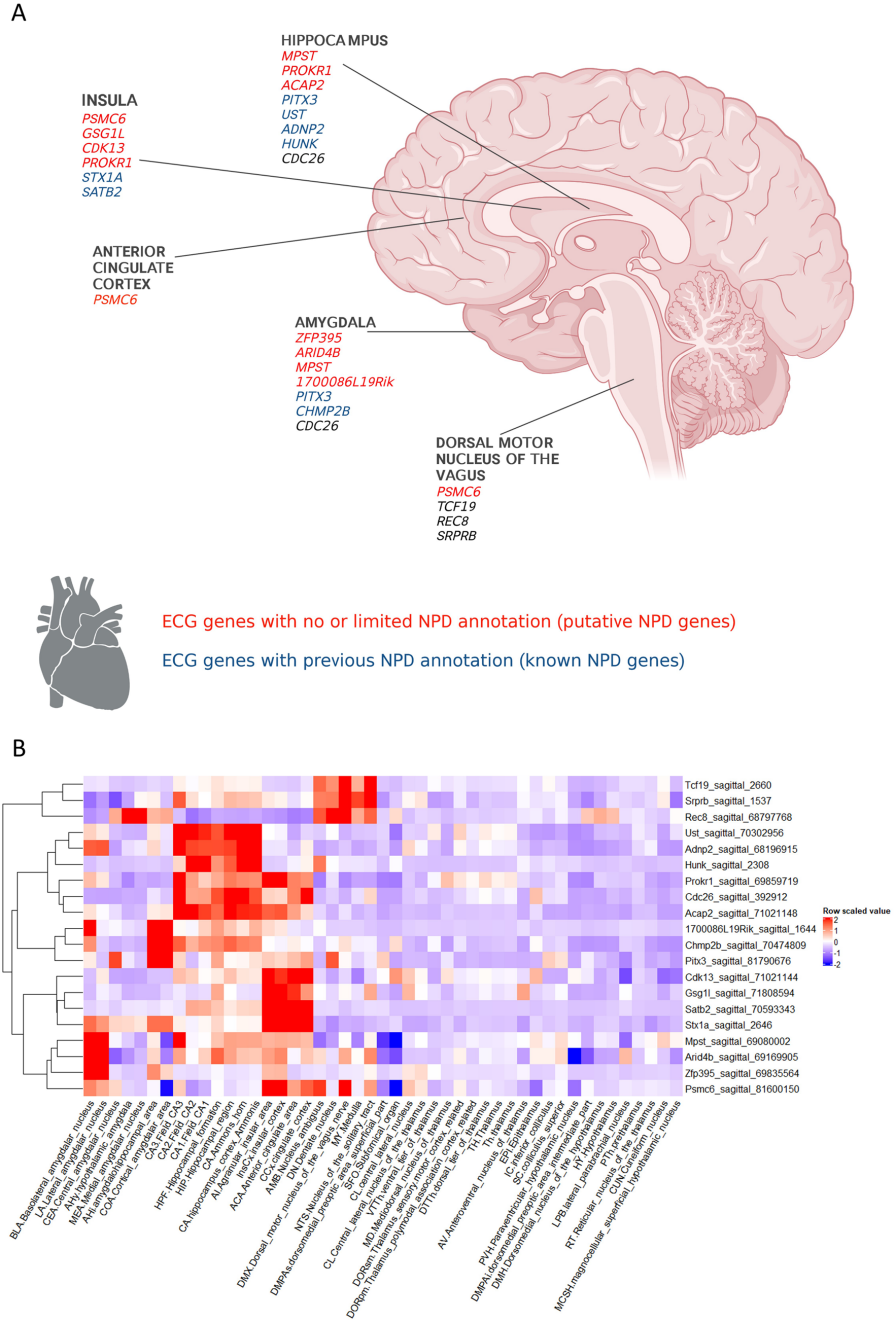
Schematized overview of established and putative neuropsychiatric disease (NPD) genes identified based on brain central autonomic network (CAN) expression clustering of cardiovascular disease risk electrocardiogram genes. **A** Depicts the established (blue text) and putative (red text) NPD genes and the CAN brain regions with the highest expression. **B** Shows a dendrogram and heat map of the top FF-ANS and FB-ANS cluster expressed ECG genes. The columns each represent an individual brain area and the rows represent each gene (z-scores calculated across rows). Red and blue reflect up and downregulated expression levels, respectively, within each brain structure relative to all the other brain structures (A was created with Biorender.com)

The clustering algorithm grouped the ECG genes into discrete clusters with similar brain region expression. For the generation of the heat maps, we used the R package ComplexHeatmap. Agglomerative hierarchical clustering by the Heatmap function (clustering_method_columns = “ward.D2” and clustering_method_rows = “ward.D2”) was applied to group mouse brain regions (columns) as well as expression energies of genes (rows) for the heat map. The genes with the most similar expression patterns are closest in proximity in the heat map with rows scaled and represented as a z-score. The red color denoted higher expression levels and the blue had lower expression levels. We included the dendrogram for the columns, where we highlighted the subtrees with numbers (C1 and 2 for Fig. S1 and C1-7 for Fig. 3A). In addition, in Fig. 3A, we annotated the x-axis with the brain regions relevant for CAN feedforward and feedback regulation of cardiovascular function. We used the silhouette method by the function fviz_nbclust from the R package factoextra to determine this optimal number of clusters.

For gene set enrichment analysis of the clusters, we used the Enrichr web server that encompasses a broad set of libraries for analysis including information from MGI Mammalian Phenotype Level 4 2021 (that incorporated IMPC multi-systemic phenotypic data from the corresponding null mouse lines), Jensen Diseases and Enrichr Submissions TF [transcription factor]-gene co-occurrence (Chen et al. 2013; Kuleshov et al. 2016). To validate the results of our clustering algorithm, we used also a multiclass support vector machine with recursive feature elimination (mSVM-RFE) to identify the top genes defining the clusters of adult brain structures (Augustin et al. 2011). This method works through R package e1071 and assesses how often the expression pattern of a respective gene correctly classifies specified brain tissues (7 classes in this case) from randomly drawn subsets of the original dataset. First, expression energies of 7 clusters C1-7 resulting from hierarchical clustering were randomly grouped into stratified four folds by using all combinations of three folds, which results in four combinations for mSVM-RFE. This grouping was completed fifty times arising in a total of 200 mSVM-RFE runs or 200 different gene selections in the end. mSVM-RFE starts with all 418 gene expression profiles (i.e. features) and ends up with 15 representing the stop condition for iterations. In each run, mSVM-RFE recursively eliminates 10% of the remaining expression values, which are not good enough for classification according to the cost function of the SVM classifier. The SVM function in R was used with default settings except the parameters type = “C-Classification”, kernel = “linear” and cost = 0.01.

### Results—clustering based on brain expression and enrichment analysis of ECG-related genes

We clustered 418 ECG genes focusing on 47 predefined brain regions recruited for feedforward or feedback CAN control responses as summarized in see “An overview of heart/brain communication via CAN—feedforward and feedback ANS control” section (Fig. 3A, clustering based on all brain areas shown in Fig. S1) (Dampney 2016). We identified two main ECG gene clusters (designated “FF-ANS” [feedforward] and “FB-ANS” [feedback]) based on relative expression within seven CAN-relevant brain structure clusters (C1-7, See Supplementary information for gene list). FF-ANS consisted of 212 ECG genes with the highest expression in mainly the amygdaloid and hippocampal subfields as well as the insular and cingulate cortical areas (highest in clusters C1, C3 and C4 with relatively lower expression in C2). The FB-ANS cluster comprised 206 genes with the highest expression in brainstem nuclei (cluster C5), more variable in feedforward brain areas (C1-4 and C6-7 hypothalamic and thalamic subnuclei).

We hypothesized that the FF-ANS expressed genes influence ECG activity by mediating effects of cognitive and emotion-related higher brain function. The FB-ANS genes, on the other hand, were potentially active in response to cardiovascular baroreflex information. Consistent with our hypothesis, the top enriched mammalian phenotype (MP) terms associated with FF-ANS genes related to behavioral brain function. These included hyperactivity, abnormal sleep behavior, decreased exploration of a new environment, decreased startle reflex and impaired sensorimotor gating [decreased prepulse inhibition] (Fig. 3B, tables of genes in Supplementary information). Preweaning lethality complete and incomplete penetrance also enriched within the FF-ANS gene set suggesting a strong essential developmental role for a subset. Confirming our initial gene selection based on ECG phenotypes, increased heart rate, shortened ST segment and shortened RR interval were also among the top enriched MP terms (Fig. 3B). The top enriched related disease within the FF-ANS gene set was “Mental Depression” (Fig. 3C) and the most significant transcription factor-gene co-occurrence were POGZ and ASH1L (Fig. 3D). Pogo transposable element derived with ZNF domain (POGZ) is the most frequently de novo mutated gene in neurodevelopmental disorder patients including ASD (De Rubeis et al. 2014; Matsumura et al. 2020; Sanders et al. 2015; Ye et al. 2015). ASH1-Like Histone Lysine Methyltransferase (ASH1L) is also a major ASD risk factor with decreased PFC levels evident in post-mortem ASD patients (Qin et al. 2021). The FF-ANS cluster is comprised therefore of cognitive and emotion-related behavior and disease genes indicating that our algorithm has gathered a gene set with potential NPD relevance.

Conversely, the FB-ANS gene set did not enrich for behavior but rather ECG-related phenotypes such as shortened RR interval, increased HR and abnormal sinus arrhythmia (Fig. 3B). Pre-weaning lethality complete penetrance was overrepresented albeit concerning fewer genes than in FF-ANS (38 genes in the case of FB-ANS while 50 genes in the corresponding FF-ANS category). There were no strong disease or transcription factor-gene co-occurrence associations (i.e. significant with adjusted *P* value [*P*adj], Fig. 3C and D). Nevertheless, “coronary artery disease” and “reflex sympathetic dystrophy” featured among the top enriched disease terms with unadjusted significance that were not significantly enriched in FF-ANS disease terms (Fig. 3C). This profile indicates that FB-ANS genes are involved in cardiovascular reflex responses. Overall, our combined hierarchical cluster and enrichment analysis identified two gene sets based on brain-region expression profiles with distinct functional and disease enrichments.

### Support vector machine inquiry and literature classification of top CAN expressed ECG genes

To validate our clustering approach, we used a support vector machine (mSVM-RFE) to pinpoint the top expressed genes defining the clusters (Augustin et al. 2011). We extracted 99 in total across both clusters, FF-ANS (55 genes) and FB-ANS (44 genes, Full list of genes in Supplementary information). We then used existing mouse and human data and literature concerning the top 5 cluster discriminating genes to confirm that the analysis gathered genes with similar functions and NPD relevance. The top FF-ANS genes were *Satb2, Stx1a, Adnp2, Ust* and *Acap2.* Both *Satb2* and *Stx1a* are highly expressed in the adult mouse ACC and insular cortices (Fig. 4A and B). The former encodes SATB homeobox 2 (SATB2) protein and the latter, syntaxin 1A. SATB2 functions during mouse brain development (Britanova et al. 2008) and both SATB2 and STX1A function in the adult brain and are associated with SCZ (Jaitner et al. 2016; Whitton et al. 2018; Wong et al. 2004). The genes *Adnp2, Ust* and *Acap2* are highly expressed in the hippocampus and *Adnp2* also in the amygdala (Fig. 4A and B). *Adnp2* encodes the activity-dependent neuroprotective protein (ADNP2), expressed during development and associated with ASD and SCZ (Dresner et al. 2011; Helsmoortel et al. 2014; Van Dijck et al. 2019). *Ust* encodes the protein uronyl 2-sulfotransferase (UST) (Nikolovska et al. 2015), which is downregulated in MDD (Oommen et al. 2021). *Acap2* encodes ArfGAP with coiled-coil, ankyrin repeat and PH domains 2 (ACAP2) protein with no prior NPD annotations. Thus, four of the top five ECG genes within CAN brain structures (FF-ANS) have previous NPD associations.

The top FB-ANS cluster genes were *Rec8, Cdk13, Srprb, Hunk* and *Tcf19.* All three of the genes *Rec8* (encoding REC8 meiotic recombination (REC8) protein), *Srprb* (encoding signal recognition particle receptor subunit beta (SRPRB) protein) and *Tcf19* (encoding Transcription Factor 19 protein) while exhibiting relatively higher brain expression in the brainstem nuclei (Fig. 4A and B) are higher expressed in other tissues (*Rec8*: testis, *Srprb*: pancreas, bones, *Tcf19*: immune cells, oesophagus, see www.proteinatlas.org). Figure 4B illustrates the distinct mouse expression pattern where the left Y-axis dendrogram shows a clear separation of these three genes from the remainder. Moreover, they do not have previous CNS disease implications and ablation did not alter behavior. The remaining two genes, *Cdk13* (encodes cyclin-dependent kinase 13 (CDK13) enzyme) and *Hunk* (encodes Hormonally Up-Regulated Neu-Associated Kinase (HUNK) protein) express in adult mouse insular/cingulate cortices and hippocampus respectively (Fig. 4A and B) as well as in other adult tissues (*Cdk13*: gastrointestinal tract, kidney, *Hunk*: multiple tissues, www.proteinatlas.org). Nevertheless, at least one of these genes plays a developmental disorder role (Hamilton and Suri 2019). In summary, the top FB-ANS cluster ECG genes do not have strong previous CNS disease associations with exceptions related to aberrant development. Furthermore, the higher relative expression of these genes outside the adult brain is consistent with the lack of brain disease connections and indicates that the clustering analysis has grouped genes with more limited adult brain expression and function and/or confined to a feedback regulatory role in cardiovascular function. This information concerning the FF-ANS and FB-ANS clusters further confirms the validity of our clustering approach to identifying potentially novel NPD-relevant genes associated with abnormal cardiovascular/autonomic function.

### Novel insights from altered HR/HRV with hyperactivity to index CAN dysregulation

Given the HR/HRV and CAN/ANS connection, we manually identified the top CAN-expressed ECG genes associated with abnormal HR/HRV (32 genes, Table 1, see Supplementary information for gene lists and 23 genes associated with altered heart rhythm QRS and QTc parameters). These enriched for the mouse phenotype “hyperactivity”, which is of interest in terms of implementing HRV as a proxy for PFC function as proposed by the Neurovisceral Integration Model (Thayer and Lane 2000). Hyperactivity is at least partially emblematic of impaired inhibitory PFC influence on inappropriate behavior and attention (Ma et al. 2005) as well as impaired parasympathetic vagal tone and sympathetic overdrive. Decision-making and impulse control ability rely on maintaining a balance between movement initiation and inhibition in response to extraneous stimuli necessitating intact PFC activity (Hardung et al. 2017). Eleven of the 32 CAN expressed genes increased HR, eight of which exhibit no or limited previous NPD annotation (Table 1). This highlights how our clustering of the HR-altering ECG genes based on CAN brain expression successfully identified known and potentially novel NPD-related genes of which we will now focus on specific examples.

**Table 1:** Top feedforward (FF) ANS expressed genes affecting electrocardiogram (ECG) activity

Gene identifier	Mouse ECG phenotype	Mouse behavioral phenotype	Human neuropsychiatric disease (NPD) association from GWAS catalog 2019 and MGI enriched terms
*Psmc6*	HR ↑ HRV↓	Hyperactivity, decreased thigmotaxis	Limited or no NPD annotations
*1700086L19Rik*	rMSSD, HRV↓	Hyperactivity	Limited or no NPD annotations
*Mpst*	PQ ↓,HR ↑,PR ↓	Decreased exploration of a novel environment, increased latency to center entry, hypoactivity	Limited or no NPD annotations
*Mdp1*	HR ↑	Increased latency to the first transition into the dark in light/dark box, decreased % prepulse inhibition	*Bipolar disorder, mood disorder*
*Gsg1l*	HR ↓ (F),HR ↑ (M),RR ↑ (F),RR ↓ (M)	Hyperactivity, decreased startle response	Limited or no NPD annotations
*Cdc26*	HR ↑	Increased prepulse inhibition	Limited or no NPD annotations
*Chmp2b*	HR, RR ↑	Abnormal freezing behavior in fear conditioning	*Tourette's syndrome or obsessive–compulsive disorder, frontotemporal dementia*
*Pitx3*	HR ↑,ST, RR, QTc ↓	Hyperactivity, Increased coping response, decreased vertical activity, decreased exploration of a novel environment	*Cognitive ability, Parkinson’s disease*
*Prokr1*	HR ↑,RR ↓, rMSSD ↑	No	Limited or no NPD annotations
*Arid4b*	HR,QRS ↑	Decreased centre time	Limited or no NPD annotations
*Zfp395*	HR ↑ST,RR ↓	Hyperactivity	Limited or no NPD annotations
*Stx1a*	PQ↑,HR↓,ST,rMSSD,RR ↑HR_TTE ↓	No	*Disease of mental health*
*Hipk3*	HR ↓,RR, HRV ↑	Decreased startle, hypoactivity, decreased prepulse inhibition, increased acoustic brainstem response thresholds	Limited or no NPD annotations
*Srsf11*	HRV ↑	Decreased prepulse inhibition	Limited or no NPD annotations
*Tnnc1*	HRV ↑	No	*Feeling worry, Bipolar disorder, Autism spectrum disorder or schizophrenia*
*Kbtbd7*	HR ↓,RR ↑	Hyperactivity, decreased thigmotaxis	Limited or no NPD annotations
*Rasgef1a*	HR ↓, RR↑	Increased vertical activity	*Recurrent major depressive disorder*
*Cap2*	HR↓,ST, rMSSD, RR, QTc ↑	No	Limited or no NPD annotations
*Bclaf1*	rMSSD ↑	Hyperactivity	Limited or no NPD annotations
*Entpd1*	rMSSD ↑	No	Limited or no NPD annotations
*4931406C07Rik*	HR ↓ rMSSD, HRV ↑	No	Limited or no NPD annotations
*Fbxl16*	ST, RR, QTc, HR_TTE ↑ HR_TTE ↓, HRV ↑	Unresponsive to tactile stimuli	*Attention deficit hyperactivity disorder*
*Atn1*	rMSSD, HRV ↑	Limb grasping, decreased exploration of novelty	Limited or no NPD annotations
*Lpin3*	HR ↓,RR ↑	No	Limited or no NPD annotations
*Dnase1l2*	HR ↓,RR ↑,LVIDd, LVIDs ↓	Abnormal vocalisation, decreased grip strength, abnormal motor capabilities/coordination/movement, hypoactivity, small superior vagus ganglion	Limited or no NPD annotations
*Spred3*	HRV ↑	Increased freezing behavior, decreased prepulse inhibition	Limited or no NPD annotations
*Grm7*	HR ↓,RR ↑	Hyperactivity, increased grip, decreased anxiety-related behavior, limb grasping	*Recurrent major depressive disorder, Clozapine-induced agranulocytosis/granulocytopenia in treatment-resistant schizophrenia, Personality traits in bipolar disorder, Major depressive disorder (broad), Panic disorder, Neurodevelopmental disorder with seizures, hypotonia, and brain abnormalities*
*Cyp27a1*	HR,QRS↓,RR ↑	No	Limited or no NPD annotations
*Agpat1*	HR ↓,rMSSD, RR, HRV ↑	No	*Clozapine-induced agranulocytosis/granulocytopenia in treatment-resistant schizophrenia, Autism spectrum disorder or schizophrenia*
*Ctsd*	ST,RR ↑	No	neuronal ceroid lipofuscinosis
*P3h1*	HR ↓,RR ↑	Decreased grip strength	Limited or no NPD annotations
*Pnmt*	QTc Dispersion, rMSSD ↑	Increased startle response, decreased exploration of novel environment, decreased breath rate during sleep	Limited or no NPD annotations

Table shows the top ECG genes with the highest expression in the cluster FF-ANS representing feedforward central autonomic network (CAN) brain structures from Allen Brain Atlas. Also shown are the ECG and behavioral phenotypes associated with the mouse knockout (KO) lines for each gene as well as the human neuropsychiatric disease annotations

*HR* heart rate, *HRV* heart rate variability, *RR* R-R interval, ST, QTc, *QRS* ST, QTc, QRS intervals in electrocardiogram (ECG), *TTE* transthoracic echocardiography, *rMSSD* root mean square of successive differences between heart beats, shaded indicates increased HR and/or decreased HRV phenotypes in the corresponding knockout mouse line in IMPC. Neuropsychiatric disease associations are italicised

Of the HR-altering CAN genes, two also decreased HRV, *Psmc6* and *1700086L19Rik*. *Psmc6* encodes the 26S protease regulatory subunit S10B protein, a part of the 19S proteasome complex involved in cellular proteome homeostasis. It is highest expressed in the mouse adult insula and ACC as well as the dorsal motor nucleus of the vagus nerve (Fig. 4A and B). While there are limited established NPD associations, there is a link, albeit not widely researched, between PSMC6 and the pre-symptomatic stage of Huntington’s neurodegenerative disease [HD (Xiang et al. 2020)]. This phenotypic pattern, therefore, indicates that hyperactivity and reduced HRV due to mouse *Psmc6* KO could translate as an early harbinger of impaired PFC activity in HD patients with *PSMC6* mutation. Behavioral alterations including decreased concentration and decision-making ability with mood disorder characterize the pre-symptomatic stage of HD (Kirkwood et al. 2001) consistent with the mouse hyperactivity phenotype with gene ablation. A similar hyperactivity/decreased HRV pattern was evident with KO of *1700086L19Rik.* This gene has no or minimal functional or NPD annotation to date however the decreased HRV/hyperactivity phenotypes seen with loss-of-function again suggest altered sympathetic/parasympathetic balance and potentially impaired PFC and CAN function (highest expression in the mouse basolateral and cortical amygdala, Fig. 4A and B). Based on phenotype similarity, IMPC predicted psychiatric disease associations including ADHD, SCZ and neurodevelopmental disorder (https://www.mousephenotype.org/data/genes/MGI:1921534). Thus, *1700086L19Rik* is a potential novel NPD-risk gene predicted by our clustering algorithm, related to CAN and autonomic dysfunction that is of interest for future detailed investigation.

Three additional CAN/FF-ANS genes revealed through our clustering analysis increased HR and caused hyperactivity on ablation in mice: *Pitx3* (Pituitary homeobox 3), *Gsg1l* (Germ Cell-Specific Gene 1-Like Protein) and *Zfp395* (zinc finger protein 395). *Pitx3* expresses in midbrain dopaminergic neurons (highest adult CAN expression in the central and cortical amygdala, Fig. 4A and B) and is involved in Parkinson’s disease pathogenesis (Le et al. 2011; Li et al. 2009). Predictions indicate that GSG1L plays a role in AMPA receptor activity regulation in the hippocampus with the highest CAN expression in the insula and ACC (Fig. 4A and B) (Gu et al. 2016). Abnormal dopaminergic and glutamatergic activity leads to CAN dysregulation and thus the associated phenotype pattern described here emphasizes the value of HR measurement as an early index of aberrant CAN activity. ZFP395 (also known as HD Gene Regulatory Region-Binding Protein 2) is located in the HD gene promotor region (Tanaka et al. 2004). There is some scant empirical data implicating this gene in HD however this phenotypic pattern of increased HR/hyperactivity and the highest expression in the basolateral and lateral amygdala (Fig. 4A and B) again suggests CAN dysregulation for further investigation related to this disease. These examples illustrate how likely dysregulation of the CAN in parallel disinhibits sympathetic drive and impairs parasympathetic vagal tonic control of HR. Thus, the combination of mouse hyperactivity and HR/HRV signifies likely CAN disruption including PFC and hippocampal impairments. The remaining CAN-expressed genes gathered by our clustering algorithm with increased HR (*Mpst, Arid4b, Mdp1, Cdc26* and *Chmp2b*), high amygdala and hippocampal expression (Fig. 4A and B) and mouse behavior phenotypes (abnormal sensorimotor gating, emotionality, Table 1) are therefore additional genes where HR likely signifies aberrant CAN function and are a mixture of known and possibly novel NPD genes of interest.

The picture for increased mouse HRV and altered behavior associated with CAN-expressed gene clustering is less clear (Table 1). Nevertheless, these cases likely involve brainstem abnormality rather than higher CAN dysfunction. For example, *Hipk3* [Homeodomain Interacting Protein Kinase 3 (HIPK3) protein] is highly expressed in the heart and the IMPC predicts a role for this gene in the ANS disease Neuropathy, Hereditary Sensory and Autonomic, Type Vi (https://www.mousephenotype.org/data/genes/MGI:1314882). Moreover, loss of the mouse genes *Spred3* [Sprouty Related EVH1 Domain Containing 3 (SPRED3) protein] and *Fbxl1* (F-box/LRR-repeat protein) increase HRV and alter emotionality requiring further investigation to determine the disease relevance.

## Summary and conclusion

Evidence implicates heart/brain axis and CAN/ANS dysfunction in NPD yet there remain gaps in understanding the genetic underpinnings as well as in delineating potential therapeutic targets. Moreover, HRV, as an index of intact CAN-ANS interaction, is a highly heritable trait and putative non-invasive proxy of higher brain function and NPD endophenotype (Golosheykin et al. 2017). Mouse multi-systemic data has the potential to yield relevant and innovative NPD therapeutic insights not feasible yet in a clinical setting. Therefore, we provided here a rationale for tackling large-scale mouse genetic data in the public domain to gain insights into heart/brain axis control in NPD. As a unique approach, our initial gene set concerned abnormal ECG and CVD risk to distinguish potential NPD genes related to abnormal CAN/ANS. To that end, we introduced a stepwise refinement of the extensive aberrant ECG gene set. Combining hierarchical clustering of regional brain expression levels and enrichment analysis of these genes, as well as support vector machine and literature classification, we determined CAN-associated genes with NPD relevance. Furthermore, we isolated a candidate gene set highly expressed in CAN brain areas that also alter HR and/or HRV (32 of the top 99 expressed genes) and a combination of known and putative NPD genes. Our in silico validation showed that the clustering algorithm has reliably predicted established NPD genes and thus application of this well-defined pipeline to new ECG gene sets will reveal potentially novel NPD-related genes. In addition, the association of specific genes with a combination of HR/HRV alterations and novelty-induced hyperactivity represents a multimodal biomarker relevant for executive brain function and NPD, useful for isolating CAN genes of interest from the large-scale mouse data.

While this was a first step and an introductory prediction based on a limited set of ECG genes, we did uncover a poorly annotated gene *1700086L19Rik* underlying a hyperactivity/decreased HRV combined phenotype with relatively high amygdala expression. The likely influence of the encoded protein on CAN amygdala activation in response to stress exposure indicates a potential NPD pathogenic role for further deep investigation. A more in-depth analysis, applicable to any elucidated genes of interest, will necessarily entail preclinical experimental validation incorporating additional genetically engineered mouse models and more targeted technologies. As a whole body gene KO yielded this initial insight, follow up studies will require more spatially- and temporally-controlled gene KO (or knock-in) in CAN brain regions using, for example, the conditional Cre/LoxP system. In this case, a brain-specific (particularly forebrain) transgenic Cre line, e.g. calcium/calmodulin-dependent protein kinase II alpha subunit (CaMKIIα)-Cre, can be implemented in concert with functional assays to ascertain whether HRV abnormality associated with specific genes is secondary to CAN dysfunction. Alternatively, viral vector approaches such as the AAV vector-based RNA interference systems can be applied to similar effect (Kim et al. 2022). Given the therapeutic potential of sympatho-vagal balance restoration, it will be advantageous to assess the efficacy of vagal nerve stimulation as well as that of other pharmacological ANS modulatory agents and exercise on behavioral and cardiovascular abnormality in these models (van Bilsen et al. 2017).

The question then is how best to translate the validated preclinical heart/brain axis genetic control findings to human NPD patients. Our analysis confirms the importance of holistic multi-systemic analysis both for NPD and CVD patients, considering the neuropsychiatric etiology of altered HR/HRV (Shivkumar et al. 2016). Should HRV be linked to NPD genetic modifications it then represents a powerful trans-diagnostic and stratification tool as well as a treatment response metric. Effective application of this knowledge to patients therefore requires availability of genomic screening and ECG data and consideration of medical and mental conditions in parallel as advocated by DSM-5 (American Psychiatric Association 2022). Preclinical precision genetic models, generated for NPD patient-specific variants, can be analysed to determine the likely efficacy of ANS interventions.

The volume of IMPC mouse gene KO data will continue to expand to encompass all protein-coding genes in the genome. One limitation of this resource is that HRV analysis is not mandatory and so not available for all genes. Moreover, this mouse high-throughput measurement provides a first indication that will be confirmed with more robust techniques such as telemetry. These limitations aside, we expect additional CAN target elucidation for which this stepwise design will identify relevant NPD genes. Moreover, similar analyses of the associated cardiac rhythmic disorder and TTE gene sets identified previously (Spielmann et al. 2022) will provide complementary information. The role of the insular cortex and heartbeat-evoked potentials is also of interest for future investigation. Heartbeats can modulate conscious perception by augmenting heartbeat-evoked neural activity and this aligns with impaired somatosensory perception related to interoception (Al et al. 2020). Consonant genetic target identification will yield anxiety and mood disorder information. Overall, the potential insights garnered from the preclinical data will include prioritization of GWAS loci as well as application to digital phenotyping, NPD diagnosis and risk stratification for precision psychiatry approaches.

## Supplementary Information

Below is the link to the electronic supplementary material.

Supplementary file1 (PDF 680 KB)

Supplementary file2 (XLSX 531 KB)
