# Supplementary material for: A rationale for considering heart/brain axis control in neuropsychiatric disease
Source: Mamm Genome. 2022 Dec 20;34(2):331–50. doi: 10.1007/s00335-022-09974-9 (PMC10290621; doi:10.1007/s00335-022-09974-9)
Supplement: Supplementary file 1 — Supplementary file1 (PDF 680 KB) [file 335_2022_9974_MOESM1_ESM.pdf]

FIGURE S1

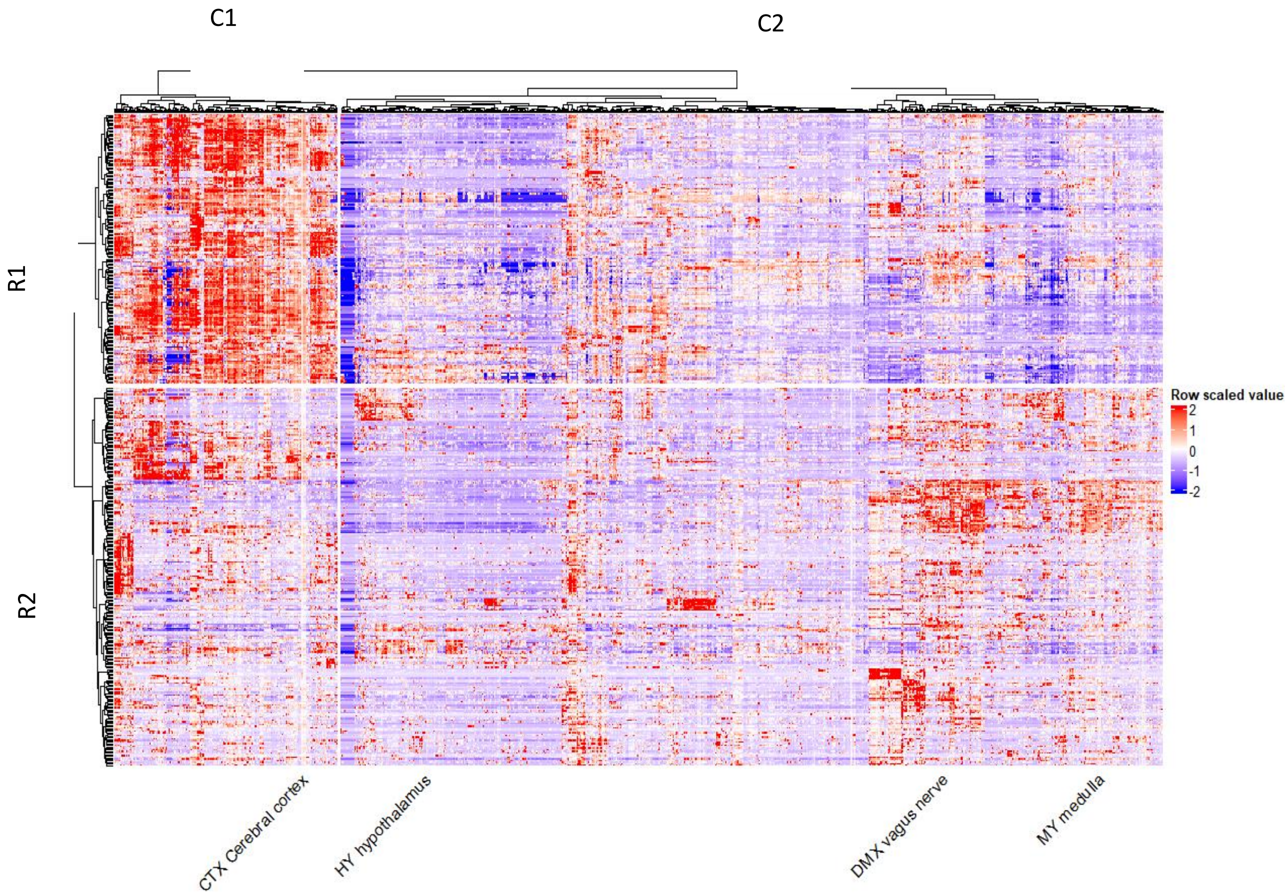

Fig. S1. The clustering analysis of all anatomical brain regions based on the **ECG gene expression** divided the genes into two groups (clusters R1 and R2) based on their expression in two brain region clusters (C1 and C2). Cluster R1 consisted of 174 ECG genes with similar expression patterns in embryonic telencephalon-derived brain structures involved in feedforward control of cardiovascular activity (cortical and hippocampal subfields, Cluster C1). These genes exhibited minimal expression in more hindbrain structures and subnuclei that modulate cardiovascular feedback control (Cluster C2). The cluster R2 consisting of 244 ECG genes exhibited lower brain expression in telencephalic brain regions (cluster C1) and higher expression in brainstem nuclei including dorsal motor nucleus of the vagus and are likely involved in cardiovascular feedback control.
